# Danger from the Bicycle

**Published:** 1886-06

**Authors:** 


### DANGER FROM THE BICYCLE.

Each new development in the mechanical arts of civilization seems to bring with it its own special disease. Even the pleasures and recreations of the student or the business man often bring, with the improvement in general health, a diseased action in some special organ which is by chance thus subject to some undue strain or irritation. The public is beginning to understand the danger of heart strain that comes to the student with the increased muscle and apparent vigor which result from the training for the college regatta.

The disease which will be induced by the use of the bicycle will not be so well understood. The horseman, after his fortieth year, is apt to show symptoms of disease of the prostate gland. It is the result of the pressure of the saddle against the gland, The pressing and the jarring create an irritation, which passes into a chronic congestion, then glandular hypertrophy, with mechanical obstruction to the free escape of urine, a bladder developing a chronic cystitis from the retained and decomposing fluid, a secondary kidney affection, and death. The extra risk of development of this line of disease to the horseman is well known. Yet the saddle which he uses affords quite a broad, secure seat. If one examines the saddle or seat of the bicycle, however, he finds a narrow support of only a few inches for the whole weight of the body. A wider saddle is not possible, as it would interfere with the free use of the feet on the pedals. The horseman, upon the broad saddle, has the additional advantage that the weight of the body rests principally upon the firm tuberosities of the ischii, while with the bicyclist, owing to the narrowness of the saddle, the weight comes upon the perineum, and is transmitted directly to the prostate gland and base of the bladder– If the horseman develops a tendency beyond that of the average of men to trouble with the prostate and urinary organs, the habitual user of the bicycle must develop to a much more marked degree the same tendency, and the result of a general use of the bicycle must be an equally general tendency to hypertrophy of the prostate with its attendant ills.—*S. California Practitioner*.

